# Supplementary material for: Host–virus association databases as tools for understanding viral spillover at varying scales
Source: PLoS Negl Trop Dis. 2026 May 18;20(5):e0013343. doi: 10.1371/journal.pntd.0013343 (PMC13193610; doi:10.1371/journal.pntd.0013343)
Supplement: Attachment — Submitted filename: Rebuttal letter.docx [file pntd.0013343.s002.docx]

Rebuttal letter

Article: " Host-virus association databases as tools for understanding viral spillover at varying scales"

We would like to begin by thanking the reviewers for their time spent on evaluating this manuscript. We explain how we have addressed the point raised below. The statement of the reviewer is presented in bold font.

1) **This appears to be a resubmission of the manuscript PNTD-D-25-01041. Please include a rebuttal letter with a response to the decision and reviewers’ comments (if applicable), detailing any revisions made to the original submission. Please upload this as a 'Response to Reviewers' file type.**

We have resubmitted this manuscript (PNTD-D-25-01065) as a research article as when we submitted it as a review (under PNTD-D-25-01041) we received the following feedback:

*Thank you for submitting your manuscript to PLOS Neglected Tropical Diseases. As with all papers, your manuscript was reviewed by members of the editorial board. Based on our assessment, we ask you to resubmit your article in the proper category, as an original article and not a review, since it includes a practical "experiment".*

Therefore, we are now submitting the manuscript as a research article under the reference number: PNTD-D-25-01065. We have resubmitted it is a research article as it includes a practical ‘experiment’.
