# Supplementary material for: Host–virus association databases as tools for understanding viral spillover at varying scales
Source: PLoS Negl Trop Dis. 2026 May 18;20(5):e0013343. doi: 10.1371/journal.pntd.0013343 (PMC13193610; doi:10.1371/journal.pntd.0013343)
Supplement: Attachment — Submitted filename: Author return letter to revisions.docx [file pntd.0013343.s004.docx]

February 11, 2026

Dear Dr. Jonsson,

I have uploaded a revised version of our manuscript, *“Host–virus association databases as tools for understanding viral spillover at varying scales”* (PNTD-D-25-01065), to the Editorial Manager portal, and it is now ready for your review.

In revising the manuscript, we carefully addressed both reviewer comments as well as the editorial feedback provided on the manuscript. In response, we added a quantitative dataset and accompanying table, revised the language throughout to clarify how this work builds on previous studies, and incorporated additional references to better situate our findings within the existing literature. All revisions were made in line with the requested edits.

Below, we have included the full set of reviewer comments followed by our responses (in **bold**). Line numbers cited refer to the version of the manuscript with tracked changes enabled. For ease of reference, we have also uploaded a clean version of the manuscript without tracked changes, representing the final revised submission.

Thank you again for your time and consideration. Please do not hesitate to contact me if you have any questions.

Kind regards,
Imogen Lindsley

*Reviewers' comments:*

Methods

Reviewer #1: The data are described clearly, and are appropriate for the study. The methods are also described clearly and appropriate for the study.

Reviewer #2: The study design and methods are clearly described, but the analytical depth is limited. The approach primarily involves filtering the VIRION database for Orthoebolavirus entries and manually extracting metadata from ~40 source studies (of which 19 are surveillance studies). While the effort to cross-check and verify metadata is useful, the scope of the dataset is too narrow to support broader inferences about database performance or spillover mechanisms.

**We agree with the reviewer that the scope of the study limits the ability to draw broader inferences about spillover mechanisms. Our intention was not to make conjectures about *Orthoebolavirus* spillover mechanisms, but rather to assess how well host–virus association databases support analyses at different scales. We have clarified this throughout the manuscript and explicitly acknowledged the limited sample size as a key limitation (line 843). We have also further situated our findings within the context of previous studies that reported similar challenges (e.g., Carlson et al. 2022; Gibb et al. 2021; Simons et al. 2023), and we now emphasise that our results mirror and support this existing body of work.**

There is no quantitative or comparative analysis included — only descriptive summaries. The so-called “experiment” mentioned in the resubmission letter refers to manual data review and does not constitute an analytical or empirical experiment. To strengthen this section, the authors could include some quantitative assessment of metadata completeness (e.g., percentage of studies reporting negative data, spatial coordinates, or detection methods). That would provide an objective measure of data quality and improve the credibility of the “fine-scale” assessment they aim to demonstrate.

**We agree with this suggestion and have now included quantitative summaries of metadata completeness. Specifically, we added a new “Dataset description” Results section that presents summary statistics for the full VIRION dataset, as well as more detailed quantitative reporting for the 40 *Orthoebolavirus* studies, including the proportion of studies reporting negative data and other key metadata fields (Lines 248-382). We removed the other table that simply extracted data from the supplementary information, as these data were already presented there and their inclusion would have been redundant (Line 404).**

Results

Reviewer #1: Yes, to all three questions.

Reviewer #2: The results section primarily summarizes what is already visible from the databases and the original studies. The figures (1–3) are descriptive — listing host species, reporting positive and negative samples, and showing sample types. These are useful visual aids but do not offer new insights or analyses.

**We acknowledge this concern and have strengthened the Results section by adding a quantitative description of database coverage and metadata completeness, rather than relying solely on descriptive figures. This includes new summary statistics and a table detailing the availability of key metadata fields, which provides additional analytical depth (Lines 248-382).**

Importantly, the number of studies (40 total, 19 with new detections) is too small to claim that the analysis tests database “utility at varying scales.” The conclusions drawn — that negative results and metadata are often missing, and that databases are better suited for large-scale rather than fine-scale analyses — are correct but already well established in the literature (e.g., Carlson et al. 2022; Gibb et al. 2021; Simons et al. 2023).

**We agree with the reviewer and have softened the language throughout the manuscript to avoid overstating the scope or novelty of our findings (see Lines 24, 44, 678, 859). We now explicitly acknowledge the small sample size (line 843) as a limitation and frame our results as supporting and extending existing evidence rather than introducing novel conclusions.**

The section also mixes interpretation and guidance (e.g., “researchers can enhance these analyses using complementary biodiversity datasets”) into what should be reported results. Separating these into results versus recommendations would improve clarity. A quantitative summary table showing the frequency of missing fields or metadata would make the results section more substantial and analytical.

**We agree and have moved interpretative statements and recommendations from the Results section into the Discussion (see Lines 656, 662, 800, 807, 817). We have also added a quantitative summary table showing the frequency of missing metadata fields, as suggested by the reviewer (see Lines 248-382).**

Conclusions

Reviewer #1: The conclusions are supported by the data, methods and results presented. I ask the authors to consider my comment on the manuscript that what this study has done is to document (not identify) the limitations of "big data" for fine-scale studies.

**We thank the reviewer and address the comment in more detail below; we have also made all suggested line edits in the attached manuscript. We thank the reviewer for this thorough feedback.**

Reviewer #2: The conclusions are reasonable and align with the descriptive findings but somewhat overstate the originality of the study. The main takeaway — that host–virus databases are valuable for broad-scale questions but limited for fine-scale spillover analyses — is valid yet not new. This has been repeatedly discussed in prior database evaluations.

**We have softened our language to reflect how the conclusions and results of this paper build on existing literature (see Lines 24, 44, 678, 859).**

The recommendations (for better metadata standards, inclusion of negative results, and consistent data sharing) are relevant and well argued but belong more to a perspective or data note than a research paper. The manuscript would therefore fit better as a Data Resource or Database Analysis Note, emphasizing transparency, metadata coverage, and lessons learned rather than as a full research article.

**We understand the reviewer’s point and have revised the manuscript to more clearly frame our conclusions as building on and supporting existing literature, rather than presenting them as novel findings (see Lines 24, 44, 678, 859). The Introduction and Discussion now explicitly situate our results within the context of current literature, and we have added additional references to reflect this context (see References 38, 56, 57, 59).**

**The paper was originally submitted as a review; however, following editorial guidance , we resubmitted it as a research article due to the original dataset derived in the Methods. In response to reviewer feedback, we have now strengthened the quantitative components of the analysis to better align with the format of a research article (see Lines 248-382 on dataset description).**

***PLOS Neglected Tropical Diseases* does not offer a Data Resource or Database Analysis Note article type, and we therefore aimed to address these concerns within the framework of a research article while maintaining transparency about the scope and limitations of the study.**

Summary and General Comments

Reviewer #1: My general sense of this manuscript is that the authors are demonstrating well, something that is already well understood by the research community--broad-scale databases with limited or inconsistent metadata are inadequate for answering fine-scale questions. This study documents that well, and has merit, and will probably be cited frequently. Nevertheless, the tone or the paper is that they are covering new ground and making a novel discovery, and this is really not the case.

**As discussed above, we have softened the language throughout the manuscript and revised multiple sections to avoid overstating novelty. We have also added additional citations to explicitly acknowledge that these issues are well established in the literature, including recent relevant work (see References 38, 56, 57, 59).**

Reviewer #2: Overall, the work is clearly written and conceptually sound but lacks analytical rigor and novelty. Expanding it with a quantitative assessment of metadata completeness, or reframing it as a data-focused paper, would strengthen its contribution and make it more appropriate for publication.

**We have added a quantitative assessment of metadata completeness and revised the framing throughout the manuscript to emphasise how this work contributes to a growing body of evidence.**
