# Supplementary material for: Host–virus association databases as tools for understanding viral spillover at varying scales
Source: PLoS Negl Trop Dis. 2026 May 18;20(5):e0013343. doi: 10.1371/journal.pntd.0013343 (PMC13193610; doi:10.1371/journal.pntd.0013343)
Supplement: Attachment — Submitted filename: Author return letter to reviewers - April.docx [file pntd.0013343.s005.docx]

April 14, 2026

Dear Dr. Jonsson,

I have uploaded a revised version of our manuscript, *“Host–virus association databases as tools for understanding viral spillover at varying scales”* (PNTD-D-25-01065R1), to the Editorial Manager portal, and it is now ready for your review.

In revising the manuscript, we carefully addressed the additional reviewer comments. In response, we refined the language, particularly in the discussion, to more clearly articulate practical recommendations for researchers and to highlight how this paper supports their implementation. All revisions were made in line with the requested edits.

Below, we have included the full set of reviewer comments followed by our responses (in **bold**). Line numbers cited refer to the version of the manuscript with tracked changes enabled. For ease of reference, we have also uploaded a clean version of the manuscript without tracked changes, representing the final revised submission.

Thank you again for your time and consideration. Please do not hesitate to contact me if you have any questions.

Kind regards,
Imogen Lindsley

PNTD-D-25-01065R1

Host-virus association databases as tools for understanding viral spillover at varying scales

Reviewers' comments:

Methods

Reviewer #1: As in the earlier version, Methods are appropriate, and explained clearly.

Reviewer #3: The objectives are clearly stated in this manuscript, as are the methods used to address the questions asked.

**No amendments to be made. Thank you.**

Results

Reviewer #1: As in the earlier version, Results seem reasonable, not unexpected.

Reviewer #3: The results are clearly presented.

**No amendments to be made. Thank you.**

Conclusions

Reviewer #1: The conclusions are better presented than in the earlier version, and are reasonable; again, not unexpected.

Reviewer #3: The conclusions are generally supported by the data.

**No amendments to be made. Thank you.**

Editorial and Data Presentation Modifications?

Reviewer #1: A couple of minor issues came to mind in this reading:
1. (Lines 460, 463) I have no idea what the term "real-time" means in the context of either of these statements. Please clarify and/or use a different term.

**We have modified our language to clarify that we are referring to “dynamic” databases, which allow continuous updates (as opposed to “static” databases) in line 471 & 472.**

2. (Line 472) The IUCN Red List quite often over-states a species' distribution; it should not be taken as a definitive distribution description of host species.

**We now discuss this limitation in line 495.**

Reviewer #3: N/A

**No amendments to be made. Thank you.**

Summary and General Comments

Reviewer #1: (Lines 476-481) These are certainly good recommendations, but it seems unlikely that they will have much or any impact on the research and database communities. Can the authors offer any more practical recommendations, such that the publication of this paper will have a practical impact, rather than just confirming the known limitations of data mining?

**We now summarise practical, actionable recommendations for researchers and database curators in lines 504-511.**

Reviewer #3: The revised manuscript by Lindsley and Juman et al. describes the use of the VIRION database for addressing questions regarding the distribution and transmission of ebolaviruses and Lassa viruses. Overall, the manuscript is well-presented and addresses an important aspect of data harvesting. A few modifications would improve the discussion section:

1. The authors discuss shortcomings in the published/deposited data. To this discussion, they could add that there can be concerns regarding false positive and false negative serology due to cross-reactivity or low sensitivity, respectively.

**We now discuss this in lines 422-424.**

2. A brief discussion of how researchers should interpret surveillance data from PCR versus serology would be useful.

**We now discuss this in lines 417-422.**
