# Supplementary material for: Host–virus association databases as tools for understanding viral spillover at varying scales
Source: PLoS Negl Trop Dis. 2026 May 18;20(5):e0013343. doi: 10.1371/journal.pntd.0013343 (PMC13193610; doi:10.1371/journal.pntd.0013343)
Supplement: Attachment — Submitted filename: PNTD-D-25-01065, reviewer comments.pdf [file pntd.0013343.s003.pdf]

PLOS Neglected Tropical Diseases

Host-virus association databases as tools for understanding viral spillover at varying scales

--Manuscript Draft--

**Manuscript Number:**	PNTD-D-25-01065
**Full Title:**	Host-virus association databases as tools for understanding viral spillover at varying scales
**Short Title:**	Using host-virus association databases to understand viral spillover
**Article Type:**	Research Article
**Keywords:**	zoonotic spillover; reservoir; metadata; host-virus interactions; Zoonotic disease; mammarenavirus lassaense; orthoebolaviruses; one Health
**Abstract:**	Large host-virus association databases are increasingly used to explore broad questions in disease ecology, particularly around host range, pathogen diversity, and the potential for spillover. While these databases have been instrumental in large-scale synthesis of host-pathogen biogeography and zoonotic risk, their potential role in addressing fine-scale questions about pathogen prevalence, maintenance, and transmission dynamics remains to be tested. In this study, we assess how different types of data, including entries in databases and the original studies they draw from, can support targeted research on zoonotic spillover. Using the Orthoebolavirus entries in the VIRION database as a case study, we evaluate the extent to which databases capture crucial contextual metadata, such as spatial and temporal resolution, negative results, and measures of viral load. Guided by a conceptual framework of factors that lead to spillover, we demonstrate that while host-virus databases are valuable for addressing high-level patterns, fine-scale investigations of spillover require specific studies with detailed epidemiological data. We conclude by offering practical recommendations for database users and managers, highlighting how these tools can be used as starting points in broader spillover research.
**Additional Information:**	
**Question**	**Response**
**Government Employee**<br><br>Are you or any of the contributing authors an employee of the United States government?<br><br>Manuscripts authored by one or more US Government employees are not copyrighted, but are licensed under a CC0 Public Domain Dedication, which allows unlimited distribution and reuse of the article for any lawful purpose. This is a legal requirement for US Government employees.<br><br>This will be typeset if the manuscript is accepted for publication.	No - No authors are employees of the U.S. government.
**Financial Disclosure**	The author(s) received no specific funding for this work.

Powered by Editorial Manager® and ProduXion Manager® from Aries Systems Corporation

Enter a financial disclosure statement that describes the sources of funding for the work included in this submission. Review the submission guidelines for detailed requirements. View published research articles from *PLOS Neglected Tropical Diseases* for specific examples.<br><br>This statement is required for submission and **will appear in the published article** if the submission is accepted. Please make sure it is accurate.<br><br>**Funded studies**<br>Enter a statement with the following details:<br>• Initials of the authors who received each award<br>• Grant numbers awarded to each author<br>• The full name of each funder<br>• URL of each funder website<br>• Did the sponsors or funders play any role in the study design, data collection and analysis, decision to publish, or preparation of the manuscript?<br><br>Did you receive funding for this work?	
**Competing Interests**<br><br>On behalf of all authors, disclose any competing interests that could be perceived to bias this work.<br><br>This statement will be typeset if the manuscript is accepted for publication.<br><br>Review the instructions link below and PLOS NTDs' competing interests policy to determine what information must be disclosed at submission.	N/A
**Data Availability**<br><br>Provide a **Data Availability Statement** in the box below. This statement should detail where the data used in this	https://github.com/viralemergence/virion

submission can be accessed. This statement will be typeset if the manuscript is accepted for publication.<br><br>Before publication, authors are required to make all data underlying their findings fully available, without restriction. Review our PLOS Data Policy page for detailed information on this policy. Instructions for writing your Data Availability statement can be accessed via the Instructions link below.	

Manuscript
Click here to access/download;Manuscript;PLOS NTD Paper Submission.docx

Host-virus association databases as tools for understanding viral spillover at varying scales

Authors and Affiliations

Imogen C Lindsley[1]*, Maya M Juman[1]*, Stephanie N Seifert[2], Rory Gibb[3], Gregory F Albery[4], Freya Jephcott[5], Olivier Restif[1]

[1]Department of Veterinary Medicine, University of Cambridge, Madingley Rd, Cambridge CB3 0ES

[2] Paul G. Allen School for Global Health, Allen Center, Washington State University, 240 Ott Rd, Pullman, WA, 99163

[3]Department of Genetics, Evolution & Environment, University College London, Darwin Building, Gower St, London WC1E 6BT

[4]Department of Zoology, Trinity College Dublin, College Green, Dublin 2, D02 PN40, Ireland

[5]Centre for the Study of Existential Risk, University of Cambridge, 16 Mill Lane, Cambridge, CB2 1SB

*these authors contributed equally to this work

Abstract

Large host-virus association databases are increasingly used to explore broad questions in disease ecology, particularly around host range, pathogen diversity, and the potential for spillover. While these databases have been instrumental in large-scale synthesis of host-pathogen biogeography and zoonotic risk, their potential role in addressing fine-scale questions about pathogen prevalence, maintenance, and transmission dynamics remains to be tested. In this study, we assess how different types of data, including entries in databases and the original studies they draw from, can support targeted research on zoonotic spillover. Using the *Orthoebolavirus* entries in the VIRION database as a case study, we evaluate the extent to which databases capture crucial contextual metadata, such as spatial and temporal resolution, negative results, and measures of viral load. Guided by a conceptual framework of factors that lead to spillover, we demonstrate that while host-virus databases are valuable for addressing high-level patterns, fine-scale investigations of spillover require specific studies with detailed epidemiological data. We conclude by offering practical recommendations for database users and managers, highlighting how these tools can be used as starting points in broader spillover research.

Author Summary

There are existing large databases of viruses and the animals that have been identified as hosts. We wanted to understand how these databases can help us study the risk of diseases spreading from animals to humans, a process known as spillover. These databases are often used to answer big-picture questions, such as which animals carry the most viruses or where new diseases might emerge. However, it's not yet clear how useful these databases are for answering more detailed questions, such as how often a virus infects a particular animal, or how it spreads between animals and people.

In this study, we looked at different kinds of data, including both large databases and the original research studies that those databases are based on. We found that databases are effective for spotting large patterns, but they often lack the detail needed for more focused investigations. To study spillover in depth, we need more specific information from individual scientific studies.

Our work offers practical suggestions for researchers and database managers on how to make better use of these tools, and how they can serve as a useful starting point for more detailed disease research.

Introduction

Large public databases provide comprehensive evidence to answer classic and contemporary research questions about complex environmental and epidemiological phenomena [1,2], serving as a powerful complement to traditional systematic reviews and meta-analyses [3]. The last 20 years have seen growing concern about emerging infectious diseases, particularly those of zoonotic origin, and the potential for climate change and other anthropogenic factors to increase the risk of outbreaks. To understand recent global trends in zoonotic pathogen dynamics, several initiatives have curated and utilised host-virus association databases, collating published records of viral detections in human or animal hosts with detailed taxonomic information, along with selected metadata (e.g., detection methods, collection date, and location). The Global Virome in One Network (VIRION) database is currently the largest open-source host-virus association database [4]. VIRION compiles known host-virus associations, which are drawn from harmonised and reconciled

aggregations of individual studies: 1) in large static databases (GMPD2, HP3, Shaw, and EID2, which together form the CLOVER database); or 2) extracted daily from a singular dynamic source (GenBank). Other databases include the recently-launched PHAROS database (pharos.viralemergence.org, March 2022), which includes real-time updates of new pathogen detections, reducing the time lag associated with studies being published and aggregated. One of the biggest benefits of PHAROS is the inclusion of negative data [5,6], which is not included in VIRION. The ZOVER database [7] is also used in disease ecology research; although its taxonomic scope is limited to bat and rodent viruses, it remains a valuable resource for addressing questions at fine spatial and temporal resolutions. Combined with cutting-edge statistical and modelling methods, these databases facilitate broad-scale research on zoonotic risk, for example, predictive studies of host status [8,9]. In contrast, there has been little research into how these databases can be used to answer specific questions about spillover and its underlying mechanisms.

Plowright et al. provide a holistic framework for zoonotic spillover research [10], splitting the spillover pathway into a "hierarchical series of barriers"—factors that inhibit the flow of a pathogen from reservoir host to recipient host (e.g., host misalignment in space and time, environmental factors that limit pathogen survival, etc.). Spillover can only occur when barrier gaps align spatially and temporally to create a transmission pathway. Identifying every barrier and potential preventative intervention for a pathogen requires integrating large amounts of information, often from multiple disciplines (e.g., ecology, virology, and clinical medicine), which can rarely be coordinated into a single research initiative. Guided by this framework, we explore the feasibility of using host-virus association databases to consolidate some of this information and address questions about different stages of the transmission pathway.

For this study, we chose two emerging zoonotic diseases of major concern in Africa as case studies: viral haemorrhagic fever caused by viruses in the *Orthoebolavirus* genus (family Filoviridae) [11], and Lassa fever, caused by *Mammarenavirus lassaense* (family Arenaviridae) [12], because they exemplify the complex challenges associated with understanding viral spillover. In the case of *o*rthoebolaviruses, there remain significant unanswered questions regarding the reservoir host(s) and transmission mechanisms of the six

currently known species in this genus [13,14,15]. These uncertainties make it an ideal candidate for exploring how existing data can, or cannot, be used to address critical gaps in our understanding of spillover dynamics. *Mammarenavirus lassaense*, while somewhat better characterised, still remains understudied in terms of host ecology and transmission patterns. The primary reservoir is thought to be wild rodent *Mastomys natalensis*; however, the contributions of other rodent taxa to *Mammarenavirus lassaense* maintenance remain unclear [16]. Importantly, these viruses are the focus of sustained surveillance efforts [17], particularly in regions where they are known to be endemic. This existing surveillance infrastructure suggests that sufficient data may be available to enable robust analysis. Focusing on these two viral taxa, we interrogated the opportunities and limitations of host-virus association databases in answering high priority questions about zoonotic emergence.

Our study is structured around three questions at narrowing scales of inquiry: *Which hosts are involved in spillover? What is the pathogen prevalence across space and time? How is the pathogen maintained and shed by the host?* These questions have been structurally adapted from the framework proposed by Plowright et al. [10], which outlines the key stages of the spillover pathway upstream of human exposure to a known pathogen. For each question, we considered the type of data necessary to produce meaningful answers, and we evaluated whether these data can be sourced from existing host-virus association databases or whether additional targeted, original research is required. While it is evident that these questions go beyond the simple fact of knowing which viruses infect which hosts (i.e., the stated purpose of host-virus association databases), our goal was to assess whether other variables and metadata contained in these databases held sufficient evidence and, if not, what barriers might prevent relevant information from being recorded. We chose to focus on VIRION as it is the largest, most comprehensive open-source, open-access database of its kind. It compiles and harmonises relevant datasets and reconciles taxonomy to a single consistent backbone, so the data are accessible and standardised. However, our goal is to draw lessons applicable to any host-pathogen database and maximise their potential to support future research on spillover.

Methods

To avoid misinterpretation in this study, we employ a commonly accepted definition of *zoonotic pathogen*, which is "a pathogen that is harboured and transmitted from vertebrate nonhuman animals to humans" [10]. A general definition of *spillover*, used here, is the

"cross-species transmission of a pathogen into a host population not previously infected" [18]. We define a reservoir source population to be "one or more epidemiologically connected populations or environments in which a pathogen can be permanently maintained and from which infection is transmitted to the target population" [19, 20].

When using VIRION or the original studies cited within VIRION to investigate *Orthoebolavirus* and *Mammarenavirus lassaense* detections, we filtered the database (accessed February 21 2025) for NCBI-resolved hosts and separately for any NCBI-resolved *Orthoebolavirus* (n = 1,764 detections) and *Mammarenavirus lassaense* (n = 876 detections) species. For *Orthoebolavirus* detections, once we produced a final filtered dataset, we then read 40 of the 42 original studies in the column "ReferenceText" and extracted metadata from each study (two studies could not be accessed). The details of all 40 papers, including dates and locations of sampling, number of individuals sampled, number of pathogens sampled for, number of species sampled, type of study, detection method, and positive and negative results are listed in S1 Appendix. Out of these 40 papers, 19 were surveillance studies, 13 were experimental studies, six were scientific review papers, and two were modelling papers. Only the 19 surveillance studies constitute new evidence of viral detection. Of the total 40 *Orthoebolavirus* studies included in VIRION, only one entry inaccurately reports the pathogen detected in the original study. This GMPD2 entry incorrectly labelled the detection of *Tai Forest ebolavirus* in a chimpanzee [21] as *Zaire ebolavirus*.

Results

Here, we outline the potential ways in which host-virus association databases can be used to answer three questions across progressively narrower scales of inquiry.

Question 1 (broad scale): Which hosts are involved in spillover?

VIRION provides a high-level overview of the known host range of a virus. A basic first requirement for identifying a reservoir source population is detecting the virus within the host. Host-pathogen association databases list all the hosts that a pathogen has been detected in by taxonomic group, an especially useful feature given that review papers only sometimes

provide full lists of known host species. The VIRION database also harmonises host species to NCBI taxonomy, allowing for standardised reporting. In our filtered dataset, there were 31 host species associated with *Orthoebolavirus* from 12 families and five mammalian orders (Table 1).

A host-virus association database is a powerful tool for identifying potential co-infections, which may influence infection intensity and dynamics within hosts. Co-infection with multiple pathogens can alter immune responses, shedding patterns, and transmission potential [22, 23]. Six of 19 *Orthoebolavirus* studies referenced in VIRION also tested for other viruses (S1 Appendix). These studies highlight patterns of viral co-circulation and guide further targeted surveillance. Data on co-infection could be systematically captured in databases to improve transparency.

Broadly, host-virus associations are useful for generating initial hypotheses about which taxa may play a role in the spillover process. While they do not provide the level of detail required to establish transmission dynamics or reservoir status, they offer a broad perspective on the potential diversity of hosts and narrow down the list of species for further investigation. In the context of *Orthoebolavirus*, this allows researchers to map out candidate hosts that could be involved at various points in the spillover chain, from reservoirs to intermediate hosts, thereby guiding more targeted surveillance efforts in the field and in museum collections.

Table 1. Host species with NCBI-resolved *Orthoebolavirus* detections and associated detection methods in VIRION. Detection method refers to all types of evidence of viral presence. See S1 Appendix for full details of each detection.

**Virus**	**Host**	**Host Genus**	**Host Family**	**Host Order**	**Host Class**	**Detection Method**
Zaire ebolavirus	*Cavia porcellus*	*Cavia*	*Caviidae*	*Rodentia*	*Mammalia*	PCR/Sequencing
Zaire ebolavirus	*Cephalophus callipygus*	*Cephalophus*	*Bovidae*	*Artiodactyla*	*Mammalia*	PCR/Sequencing
Bombali ebolavirus	*Chaerephon pumilus*	*Chaerephon*	*Molossidae*	*Chiroptera*	*Mammalia*	PCR/Sequencing

Bundibugyo ebolavirus	*Chlorocebus sabaeus*	*Chlorocebus*	*Cercopithecidae*	*Primates*	*Mammalia*	PCR/Sequencing
Reston ebolavirus	*Cynopterus sphinx*	*Cynopterus*	*Pteropodidae*	*Chiroptera*	*Mammalia*	Serology
Zaire ebolavirus	*Eidolon helvum*	*Eidolon*	*Pteropodidae*	*Chiroptera*	*Mammalia*	PCR/Sequencing; Serology
Zaire ebolavirus	*Epomops franqueti*	*Epomops*	*Pteropodidae*	*Chiroptera*	*Mammalia*	PCR/Sequencing
Zaire ebolavirus	*Gorilla beringei*	*Gorilla*	*Hominidae*	*Primates*	*Mammalia*	PCR/Sequencing
Zaire ebolavirus	*Gorilla gorilla*	*Gorilla*	*Hominidae*	*Primates*	*Mammalia*	Isolation/Observation; PCR/Sequencing
Reston ebolavirus	*Hippopotamus pomona*	*Hippopotamus*	*Hippopotamidae*	*Chiroptera*	*Mammalia*	Serology
Bundibugyo ebolavirus; Sudan ebolavirus; Tai forest ebolavirus; Zaire ebolavirus; Reston ebolavirus	*Homo sapiens*	*Homo*	*Hominidae*	*Primates*	*Mammalia*	Isolation/Observation; PCR/Sequencing; Serology
Zaire ebolavirus	*Hypsignathus monstrosus*	*Hypsignathus*	*Pteropodidae*	*Chiroptera*	*Mammalia*	PCR/Sequencing
Reston ebolavirus	*Macaca fascicularis*	*Macaca*	*Cercopithecidae*	*Primates*	*Mammalia*	Isolation/Observation
Zaire ebolavirus	*Macaca mulatta*	*Macaca*	*Cercopithecidae*	*Primates*	*Mammalia*	PCR/Sequencing
Zaire ebolavirus	*Micropteropus pusillus*	*Micropteropus*	*Pteropodidae*	*Chiroptera*	*Mammalia*	PCR/Sequencing; Serology
Reston ebolavirus	*Miniopterus schreibersii*	*Miniopterus*	*Vespertilionidae*	*Chiroptera*	*Mammalia*	Serology
Bombali ebolavirus; Zaire ebolavirus	*Mops condylurus*	*Mops*	*Molossidae*	*Chiroptera*	*Mammalia*	PCR/Sequencing; Serology
Bombali ebolavirus	*Mops pumilus*	*Mops*	*Molossidae*	*Chiroptera*	*Mammalia*	PCR/Sequencing
Zaire ebolavirus	*Mus setulosus*	*Mus*	*Muridae*	*Rodentia*	*Mammalia*	PCR/Sequencing
Zaire ebolavirus	*Myonycteris torquata*	*Myonycteris*	*Pteropodidae*	*Chiroptera*	*Mammalia*	PCR/Sequencing; Serology
Reston ebolavirus	*Myotis ricketti*	*Myotis*	*Vespertilionidae*	*Chiroptera*	*Mammalia*	Serology

Zaire ebolavirus, Tai forest ebolavirus	*Pan troglodytes*	*Pan*	*Hominidae*	*Primates*	*Mammalia*	Isolation/Observation; PCR/Sequencing; Serology
Reston ebolavirus	*Pipistrellus pipistrellus*	*Pipistrellus*	*Vespertilionidae*	*Chiroptera*	*Mammalia*	Serology
Zaire ebolavirus	*Pongo pygmaeus*	*Pongo*	*Hominidae*	*Primates*	*Mammalia*	Serology
Reston ebolavirus	*Rhinolophus alitus*	*Rhinolophus*	*Rhinolophidae*	*Chiroptera*	*Mammalia*	Serology
Zaire ebolavirus	*Rousettus aegyptiacus*	*Rousettus*	*Pteropodidae*	*Chiroptera*	*Mammalia*	PCR/Sequencing; Serology
Reston ebolavirus	*Rousettus amplexicaudatus*	*Rousettus*	*Pteropodidae*	*Chiroptera*	*Mammalia*	Serology
Reston ebolavirus; Zaire ebolavirus	*Rousettus leschenaultii*	*Rousettus*	*Pteropodidae*	*Chiroptera*	*Mammalia*	Serology
Reston ebolavirus	*Scotophilus kuhlii*	*Scotophilus*	*Vespertilionidae*	*Chiroptera*	*Mammalia*	Serology
Reston ebolavirus; Zaire ebolavirus	*Sus scrofa*	*Sus*	*Suidae*	*Artiodactyla*	*Mammalia*	Isolation/Observation; PCR/Sequencing
Zaire ebolavirus	*Suncus murinus*	*Suncus*	*Soricidae*	*Eulipotyphla*	*Mammalia*	PCR/Sequencing

As a comparison, there are 22 host species associated with *Mammarenavirus lassaense* in the filtered VIRION dataset. Many of the host taxa included in VIRION reflect laboratory models including the guinea pig (*Cavia porcellus*) and the crab-eating macaque (*Macaca fascicularis*). Several other host taxa associated with *Mammarenavirus lassaense* in VIRION likely represent serological cross-reactivity from related mammarenaviruses outside the geographic distribution of *Mammarenavirus lassaense* including Asiatic bandicoot rats (genus *Bandicota*). Databases like ZOVER incorporate relevant metadata, thanks in part to the efforts of researchers. Simons et al. completed a systematic review and synthesised data from all published surveillance for *Mammarenavirus lassaense* in African rodents [12]. This revealed credible reports of viral associations with 12 rodent species and two additional genera (Figure 1).

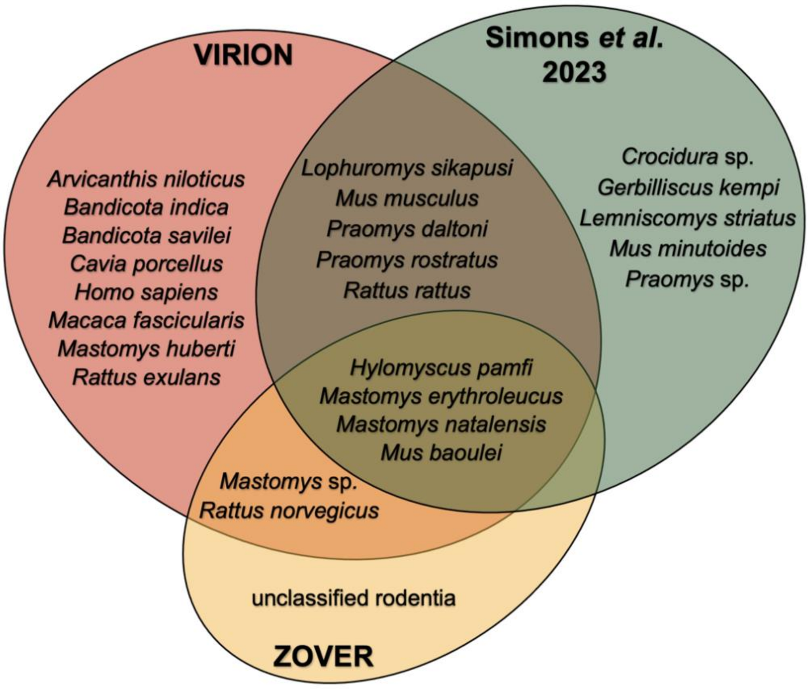

Figure 1: *Mammarenavirus lassaense* hosts as reported in VIRION, ZOVER, and Simons et al. [12].

This illustrates two key points: firstly, understanding the system of interest is critical for interpretation of aggregated host-virus association data; second, researchers should carefully consider whether their approach is to infer all possible hosts (e.g., species susceptible to infection) or the most likely species to serve as reservoirs in the wild.

Question 2 (medium scale): What is the pathogen prevalence across space and time?

Plowright et al. highlight how infection prevalence is key to determining the distribution and intensity of infection in time and space [10]. To answer medium-scale questions, such as understanding *Orthoebolavirus* prevalence across space and time, researchers require more

detailed data than what is reported in VIRION. Quantifying prevalence requires both positive and negative test results, as well as robust spatial and temporal metadata.

Negative results provide necessary context for interpreting where and when a pathogen has *not yet* been detected. Host-virus association databases theoretically allow for comparisons of the number of individuals tested for a particular virus and the total number that test positive. This positivity rate provides an estimate of pathogen prevalence within a population. However, this is only possible if negative results are reported alongside positive results. The Han et al. [8] and Schmidt et al. [24] *Orthoebolavirus*-specific datasets include this information, though presence-only databases (e.g., VIRION) do not. For each of the 19 *Orthoebolavirus* surveillance studies in VIRION, we manually extracted the number of samples collected and the subsequent proportions of positive, negative, or inconclusive results (Fig. 2). Obtaining these data required consulting the original studies, where the information was often reported in supplementary materials and consequently harder to track down.

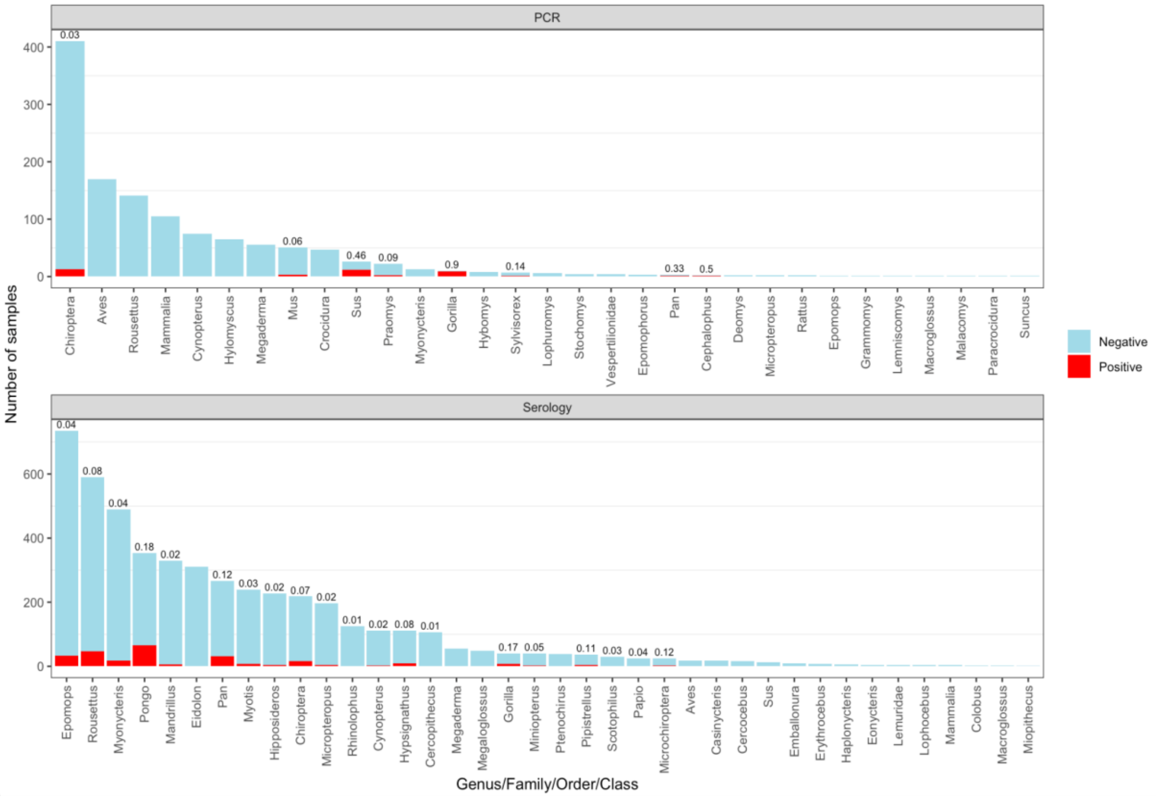

Figure 2: Positive and negative non-human samples reported in the 21 original *Orthoebolavirus* surveillance studies referenced in the filtered VIRION dataset. Numbers on top of bars indicate positivity rates for taxonomic groups with at least one positive sample.

Samples were categorised by genus or the next finest taxonomic resolution available. Viral isolation samples were not reported, as isolation is typically only attempted on PCR-positive samples. Results that were not positive were noted as negative or inconclusive following Han et al. [8], due to many studies not specifying result details.

To estimate variations in pathogen prevalence across space and time, GenBank stands out as the most reliable resource among the databases integrated into VIRION. While VIRION includes collection date for GenBank entries, spatial and temporal metadata are inconsistently reported for other contributing databases such as EID2, HP3, Shaw, and GMPD2. Our manual review of 19 *Orthoebolavirus* surveillance studies in VIRION revealed missing or incomplete spatiotemporal information in the database, limiting the usefulness of VIRION for tracking consistent viral detections across host species, time, and locations (S1 Appendix). For example, Pourrut et al. report repeated *Orthoebolavirus zairense* antibody detections in *Myonycteris torquata* across multiple years and locations [25], which were not each separately captured in VIRION. GenBank, in contrast, consistently reports country-level data and includes specific geolocation for approximately 20% of entries, with GMPD2 reporting coordinates for ~98% of its records. GenBank also includes collection dates for the majority of entries, though the level of precision varies from specific dates to year only. This is particularly important when investigating seasonal trends, such as fluctuations in bat shedding associated with reproductive cycles [26, 27, 28]. However, a limitation of GenBank is its lack of serological data, which can help infer prior exposure in potential host taxa in lieu of detecting pathogen genetic material; this is especially relevant for viruses with low-amplitude epidemic curves.

Despite a paucity of pre-2014 data, GenBank currently contains over 4,000 *Orthoebolavirus* sequences, including 332 non-human entries, offering a stronger foundation for spatial and temporal analysis than most aggregated databases. While fine-scale geographic data are ideal, especially for fragmented host populations where virus presence may vary across ranges [29, 30], even coarse data from GenBank may improve our understanding of exposure risk and transmission dynamics. Researchers can enhance these analyses using complementary biodiversity datasets such as the IUCN Red List, GBIF, and the Living Planet Index, which provide detailed host distribution data for modelling pathogen spillover risk.

For *Mammarenavirus lassaense*, in contrast, more progress has been made toward making such data accessible. Critically, work by Simons et al. resulted in an aggregate database with fine scale spatial and temporal resolution as well as inclusion of negative testing data, allowing for a true evaluation of sampling effort across host taxa [12]. These data synthesis efforts enable the investigation of finer-scale questions across space and time. This effort underscores the importance of depositing data in common databases at publication to enhance accessibility and repeatability of publicly funded research (FAIR principles) [31].

If a species is reported to harbour a virus across space and time, it may indicate that the host is a persistent source of infection. Platforms like PHAROS are working to include negative results in real-time, and genetic sequence repositories like GenBank include temporal and geographic data that help contextualise prevalence patterns. These resources enable more comprehensive analysis of pathogen prevalence across both time and space, particularly in systems where surveillance and data sharing are more advanced.

Question 3 (fine scale): How is the pathogen maintained and shed by the host?

Plowright et al. suggest that pathogen release is an important bottleneck in the spillover pathway, whereby it is a barrier that permits or constrains the flow of pathogens from one species to another [10]. They highlight how the release of a pathogen is linked to its "presence and viability in relevant tissues," which has downstream impacts on human exposure. Questions about the shedding and maintenance of a pathogen by individual host species require fine-grained data and can only be addressed through detailed epidemiological or experimental studies designed specifically to capture these dynamics. This careful and thorough investigation is particularly critical in unraveling the sources of unknown disease outbreaks, even when a certain zoonotic origin is suspected or presumed from the outset [32].

Data on viral load and tissue tropism are critical for answering these questions, as they provide clues about infectiousness, routes of transmission, and potential reservoir status. The risk of human-to-human *Orthoebolavirus zairense* transmission increases with viral load, which is greatest in the later stages of illness and immediately after death [33]. Viral load is defined as the quantity of virus in a volume of fluid at a given time and is quantified by the number of PCR cycles (Ct value) required to achieve detection [34]. For meaningful comparisons, RNA extraction and qRT-PCR procedures must be run with a standard curve [35]. However, Ct values only measure the amount of genetic material for the qPCR target

and may reflect gene expression or genome copies, therefore not directly correlating to the amount of infectious virus present. Direct measurements of infectious virus, such as plaque assays or $TCID_{50}$, are more informative for assessing transmission potential [36]. Unfortunately, information on viral load is rarely available in standard surveillance datasets or even in many original pathogen detection studies. Most of the 19 *Orthoebolavirus* surveillance studies referenced in VIRION lack the resolution needed to assess shedding dynamics, as PCR detections are typically not accompanied by quantitative viral load data.

Contextual information on sample type was not found in any of the host-virus association databases we assessed. However, we obtained the sample sources from the 19 *Orthoebolavirus* surveillance studies, excluding human entries, referenced in VIRION. These studies included 10,219 detection attempts, and sample sources were reported for all of them (Fig. 3). Reporting the sources of positive as well as negative samples is also useful in understanding the rate and mechanism of viral shedding within species (e.g., differences across age, sex, and seasons).

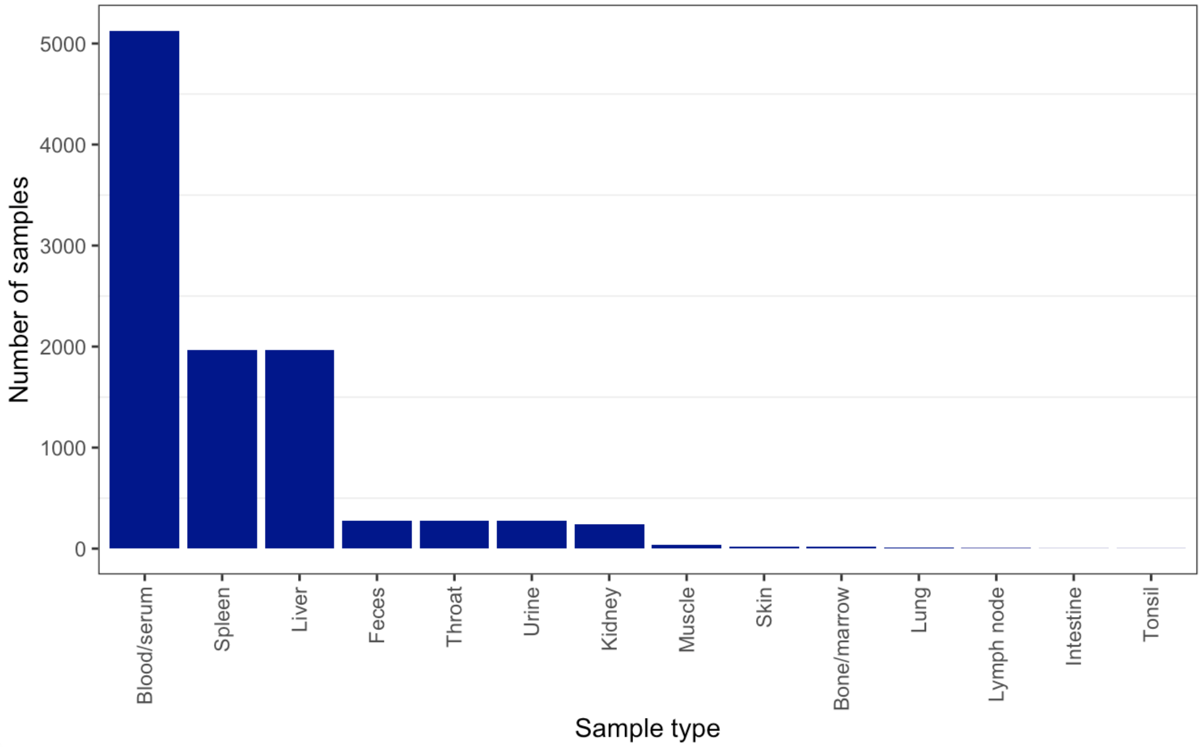

Figure 3: Sample types of all *Orthoebolavirus* entries in VIRION, extracted from the original studies and including all detection methods.

However, for certain well-studied viruses such as influenza, comprehensive epidemiological databases do exist and include fine-scale clinical and virological data, making it possible to assess maintenance and shedding patterns. This includes the World Animal Health Information System [37], WHISPers [38], and government outbreak reports, such as those published by the UK Animal and Plant Health Agency. However, for most zoonotic pathogens, particularly those that are rare or emerging, such fine-grained data remain limited.

Discussion

Considerations for database users

When using host-pathogen association databases for understanding spillover, it is crucial to clearly define the research question and consider the scale at which that question is posed. The scale, whether broad or narrow, will influence the type of data sources that are most appropriate. For broad-scale questions, particularly those concerning well-characterised and extensively studied pathogens such as influenza, host-virus databases can be immensely useful. These resources compile a vast amount of information from multiple studies, making it easier to identify general patterns, trends, and associations across different host species, time periods, or geographic regions. In contrast, when investigating more specific or fine-scale questions, such as those focused on particular host populations, local transmission dynamics, or under-studied pathogens, researchers must go beyond databases and analyse original data from the primary literature. In such cases, databases still play an important role by pointing researchers towards relevant studies, but they should not be relied upon as the ultimate source of information.

It is also essential to approach all data, whether from databases or original studies, with a critical mindset. Both sources are affected by biases and gaps, such as uneven geographic and taxonomic sampling, varying methodology, or publication bias. Researchers have begun addressing these biases in studies using both data in VIRION [39, 40] and data not in VIRION [40]; however, many biases are not currently accounted for in host-virus association databases. Below, we describe three important biases and illustrate them with findings from our study.

1. Host taxonomic bias: Focusing on a single species or taxonomic group may perpetuate a biased belief that it plays a prominent role in spillover (e.g., as a reservoir or intermediate host) despite insufficient evidence [42]. For example, there is a well-documented "bat bias" in efforts to identify the reservoir of *Orthoebolavirus* [43]. Although conclusive evidence remains elusive [15, 44], fruit bats are disproportionately represented in databases. This is partly due to the lack of negative results in presence-only datasets like VIRION, making it difficult to determine whether observed patterns reflect true distribution or sampling effort. The first direct evidence pointing to bats as reservoir hosts of *Orthoebolavirus* was published by Leroy et al. [45], when viral RNA and antibodies were detected in three species of wild fruit bats (family Pteropodidae). This drove an enormous sampling bias towards fruit bats [13, 46]. Five times as many fruit bat individuals have been sampled for filoviruses than insectivorous bats, even though fruit bats comprise only ~16% of global bat biodiversity [8] and just 12% of the 266 bat species in sub-Saharan Africa [47]. If this "bat bias" is unaccounted for, the involvement of fruit bats in filovirus spillover might be over-interpreted [43].

2. **Sampling and methodological biases:** Non-lethal sampling methods, such as blood, urine, faeces, and saliva collection, are often preferred for ethical and practical reasons, especially in small animals. Orthoebolaviruses can cause high levels of viremia, with viral RNA loads exceeding 10^{10} genome copies in nonhuman primates [48] and greater than 10^{6} viral RNA copies in Angolan free-tailed bats [49]. Consequently, blood is the most common sample type in *Orthoebolavirus* detection studies (37% of the original studies referenced in VIRION), while organ or tissue samples are underrepresented. Blood has been heavily sampled due in part to the abundance of serological studies, as well as RNA detection attempts (as orthoebolaviruses are able to infect cells circulating in blood, unlike other RNA viruses). Within the serological detection attempts, different serological assays have varying levels of specificity (due to cross-reactivity) and can test for different types of antibodies (e.g., IgG vs. IgM antibodies), hindering direct comparisons between studies. These challenges are further compounded by the lack of species-specific secondary antibodies for bats which limits assay sensitivity and specificity and often requires the use of heterologous reagents with unknown cross-reactivity (e.g., anti-mouse or proteins A and G). Additionally, bats infected with filoviruses do not appear to generate neutralising antibodies [50] which precludes the use of virus neutralization assays as the "gold standard" in serological surveillance for orthoebolaviruses in bats. The majority of the 141 host-virus associations

within the 16 studies were detected by enzyme-linked immunosorbent assay (ELISA, n = 133) and the remainder were detected by indirect fluorescent antibody test (IFA, n = 8) (see S1 Appendix). Viral isolation, which requires a cold chain and maximum containment biosafety facilities, is also rarely attempted, leading to further skew in detection methods.

3. **Geographical bias:** Surveillance studies are often conducted in response to outbreaks, resulting in an overrepresentation of data from affected areas where pathogen prevalence may be unusually high. Of the 19 *Orthoebolavirus* studies in VIRION, 14 were cross-sectional animal studies, and eight of these were carried out in the same location as an Ebola outbreak [51, 52, 53, 45, 54, 55, 25, 56].

These issues significantly affect the reliability and representativeness of the data being used, potentially leading to skewed interpretations if not properly accounted for. Therefore, researchers must be cautious and transparent about these limitations in their analyses and conclusions. Thoughtful and informed use of databases, combined with careful engagement with primary literature, is key to conducting rigorous and meaningful research on host-pathogen interactions.

Higher-level considerations for database curators, researchers, and funders

A fundamental issue is that for many pathogens, particularly those that are rare or emerging, the data required to answer key questions about spillover simply do not exist. In some cases, relevant information may have been collected, but is paywalled, poorly indexed, or not digitised at all. This presents a serious barrier to progress, particularly when studying high-consequence pathogens such as orthoebolaviruses, for which data are sparse and fragmented.

If we are to improve our capacity to understand and predict zoonotic spillover, two critical priorities must be addressed. First, there must be greater investment in data acquisition. This includes expanding and sustaining disease surveillance efforts in both human and animal populations, particularly in regions that are underrepresented in current datasets. Enhanced surveillance will help fill major data gaps and provide the foundational information needed to support robust analyses.

Second, there is a pressing need for improved data sharing, aggregation, and curation [57]. Detailed research relies on the publication of negative results as well as metadata on sample

type, collection site, collection date, co-infection status, detection method, and viral load. This depends not only on infrastructure and funding, but also on a cultural shift within the research community. Scientists must be encouraged and incentivised to submit their data to shared platforms, including real-time databases that accept both positive and negative results. An example of such a resource is PHAROS, which has significant potential but remains underutilised. By contributing data in a timely and transparent manner, researchers can help build more complete and usable datasets that benefit the entire field.

Addressing these challenges will require coordinated efforts from across the scientific ecosystem, including researchers, journal editors, database managers, and funding bodies. Without meaningful improvements in both data availability and accessibility, our capacity to answer even the most basic questions about pathogen spillover will remain limited, particularly for the rare but potentially devastating viruses that pose the greatest threats to global health.

Conclusion

In summary, host-virus association databases such as VIRION, and the datasets it draws from (e.g., GenBank), can provide valuable insights into broad-scale questions about viral spillover, particularly those concerning general host range or high-level patterns of host associations. These databases are powerful tools for large-scale synthesis, enabling systematic reviews or meta-analyses. However, we identified limitations in their current suitability as primary sources of data for fine-scale questions about spillover dynamics, such as pathogen maintenance, shedding patterns, or spatial and temporal variation in prevalence, which require detailed, context-rich data. Major gaps—in the databases themselves as well as the original surveillance studies—include a lack of negative results, a lack of precise metadata, and methodological inconsistency. We therefore recommend that, firstly, host-virus databases be used primarily as entry points for locating GenBank data or identifying key surveillance studies, rather than as final, definitive sources of data for spillover research. Secondly, database curators should seek to extract and incorporate additional contextual metadata from original studies. Thirdly, researchers who collect and publish primary data on viral detection should share complete methods and results to ensure maximum value for the wider scientific and medical community. Finally, users must consider the inherent limitations

and biases within these datasets. When applied appropriately, these databases can streamline the early stages of zoonotic spillover research, but finer-scale understanding necessitates targeted surveillance studies.

References

1. Leonelli S. Scientific Research and Big Data. In: Zalta EN, editor. The Stanford Encyclopedia of Philosophy [Internet]. Summer 2020 Edition. Available from: https://plato.stanford.edu/archives/sum2020/entries/science-big-data/
2. Runting RK, Phinn S, Xie Z, Venter O, Watson JEM. Opportunities for big data in conservation and sustainability. Nat Commun. 2020;11(1):2003.
3. Spake R, Matthews TJ, McInerny GJ, Callaghan CT. Detecting thresholds of ecological change in the Anthropocene. Ecol Evol. 2022;12(9):e9255. doi:10.1002/ece3.9255.
4. Carlson CJ, Carlson CJ, Gibb RJ, Albery GF, Brierley L, Connor RP, Dallas TA, Eskew EA, Fagre AC, Farrell MJ, Frank HK, Muylaert RL, Poisot T, Rasmussen AL, Ryan SJ, Seifert SN. The Global Virome in One Network (VIRION): an Atlas of Vertebrate–Virus Associations. mBio. 2022 Mar 1;13(2):e02985-21. doi:10.1128/mbio.02985-21.
5. Stevens T, Zimmerman R, Albery G, Becker DJ, Kading R, Keiser CN, Khandelwal S, Kramer-Schadt S, Krut-Landau R, McKee C, Montecino-Latorre D, O'Donoghue Z, Olson SH, Poisot T, Robertson H, Ryan SJ, Seifert S, Simons D, Vicente-Santos A, Wood C, Graeden E, Carlson CJ. A minimum data standard for wildlife disease research. EcoEvoRxiv. 2024 Oct 4;2024.10.04. doi:10.32942/x2js5d.
6. Clancey E, Nuismer SL, Seifert SN. Using serosurveys to optimize surveillance for zoonotic pathogens. bioRxiv. 2024 Oct 4;2024.02.22.581274v3. doi:10.1101/2024.02.22.581274.

7. Zhou S, Liu B, Han Y, et al. ZOVER: the database of zoonotic and vector-borne viruses. Nucleic Acids Res. 2022;50(D1):D943–9. doi:10.1093/nar/gkab862.
8. Han BA, et al. Undiscovered Bat Hosts of Filoviruses. PLoS Negl Trop Dis. 2016;10(7):e0004815. doi:10.1371/journal.pntd.0004815.
9. Tonelli A, Blagrove MSC, Wardeh M, Di Marco M. A framework to predict zoonotic hosts under data uncertainty: a case study on betacoronaviruses. Methods in Ecology and Evolution. 2025;16(3):611–24. doi:10.1111/2041-210X.14500.
10. Plowright RK, et al. Pathways to zoonotic spillover. Nat Rev Microbiol. 2017;15(8):502–10. doi:10.1038/nrmicro.2017.45.
11. Biedenkopf N, Bukreyev A, Chandran K, et al. Renaming of genera Ebolavirus and Marburgvirus to Orthoebolavirus and Orthomarburgvirus. Arch Virol. 2023;168:220. doi:10.1007/s00705-023-05834-2.
12. Simons D, Attfield LA, Jones KE, Watson-Jones D, Kock R. Rodent trapping studies as an overlooked information source for understanding endemic and novel zoonotic spillover. PLoS Negl Trop Dis. 2023 Jan 23;17(1):e0010772. doi:10.1371/journal.pntd.0010772.
13. Leendertz SAJ, et al. Assessing the evidence supporting fruit bats as the primary reservoirs for ebola viruses. EcoHealth. 2016;13(1):18–25. doi:10.1007/s10393-015-1053-0.
14. Olival KJ, Hayman DTS. Filoviruses in bats: current knowledge and future directions. Viruses. 2014;6(4):1759–88. doi:10.3390/v6041759.
15. Sundaram M, Gottdenker NL, Stephens PR. Where is the elusive primary ebolavirus reservoir and how do we find it? BioScience. 2025 Apr 27;73(5):1–10. doi:10.1093/biosci/biaf050.
16. Olayemi A, Cadar D, Magassouba N, Obadare A, Kourouma F, Oyeyiola A, et al. New hosts of the Lassa virus. Sci Rep. 2016 May 3;6:25280. doi:10.1038/srep25280. PMID:27140942.
17. Schuh AJ, Amman BR, Towner JS. Filoviruses and bats. Microbiol Aust. 2017;38(1):12. doi:10.1071/ma17005.
18. Wells K, Clark NJ. Host specificity in variable environments. *Trends Parasitol*. 2019;35(6):452–65. doi:10.1016/j.pt.2019.04.001.
19. Haydon DT, Cleaveland S, Taylor LH, Laurenson MK. Identifying reservoirs of infection: a conceptual and practical challenge. *Emerg Infect Dis.* 2002 Dec;8(12):1468–73. doi:10.3201/eid0812.010317.

20. Viana M, Mancy R, Biek R, Cleaveland S, Cross PC, Lloyd-Smith JO, et al. Assembling evidence for identifying reservoirs of infection. *Trends Ecol Evol.* 2014 May;29(5):270–9. doi:10.1016/j.tree.2014.03.002.
21. Formenty P, et al. Ebola virus outbreak among wild chimpanzees living in a rain forest of Cote d'Ivoire. *J Infect Dis*. 1999;179(Suppl 1):120–6. doi:10.1086/514296.
22. Plowright RK, Becker DJ, McCallum H, Manlove KR. Sampling to elucidate the dynamics of infections in reservoir hosts. *Philos Trans R Soc Lond B Biol Sci.* 2019 Aug 12;374(1782):20180336. doi:10.1098/rstb.2018.0336.
23. Ruiz-Aravena M, et al. Ecology, evolution and spillover of coronaviruses from bats. Nat Rev Microbiol. 2021. doi:10.1038/s41579-021-00652-2.
24. Schmidt JP, et al. Ecological indicators of mammal exposure to Ebolavirus. Philos Trans R Soc Lond B Biol Sci. 2019;374(1782):20180337. doi:10.1098/rstb.2018.0337.
25. Pourrut X, et al. Large serological survey showing cocirculation of Ebola and Marburg viruses in Gabonese bat populations, and a high seroprevalence of both viruses in Rousettus aegyptiacus. BMC Infect Dis. 2009;9:159. doi:10.1186/1471-2334-9-159.
26. Calisher CH, et al. Bats: Important reservoir hosts of emerging viruses. Clin Microbiol Rev. 2006;19(3):531–45. doi:10.1128/CMR.00017-06.
27. Amman BR, et al. Seasonal Pulses of Marburg Virus Circulation in Juvenile Rousettus aegyptiacus Bats Coincide with Periods of Increased Risk of Human Infection. PLoS Pathog. 2012;8(10):e1002877. doi:10.1371/journal.ppat.1002877.
28. Juman MM, Gibson L, Suu-Ire RD, et al. Ecological and reproductive cycles drive henipavirus seroprevalence in the African straw-coloured fruit bat (Eidolon helvum). Ecol Evol. 2024;14(11):e70555. doi:10.1002/ece3.70555.
29. Pulliam HR. Sources, sinks and population regulation. The American Naturalist. 1988 Nov;132(5):652–61. Stable URL at JSTOR.
30. Lloyd-Smith JO, Schreiber SJ, Kopp PE, Getz WM. Superspreading and the effect of individual variation on disease emergence. Nature. 2005;438(7066):355–9. doi:10.1038/nature23088.
31. Wilkinson MD, Dumontier M, Aalbersberg IJ, Appleton G, Axton M, Baak A, et al. The FAIR Guiding Principles for scientific data management and stewardship. *Sci Data*. 2016 Mar 15;3:160018. doi:10.1038/sdata.2016.18

32. Jephcott FL. Propagating visions of a forest reservoir: a supposed zoonotic outbreak in the Brong-Ahafo Region of Ghana. Med Anthropol Q. 2023;37(1):5–24. doi:10.1080/01459740.2023.2166411.
33. Towner JS, Rollin PE, Bausch DG, Sanchez A, Crary SM, Vincent M, et al. Rapid diagnosis of Ebola hemorrhagic fever by reverse transcription-PCR in an outbreak setting and assessment of patient viral load as a predictor of outcome. J Virol. 2004 Apr;78(8):4330–41.
34. Li J, et al. Age and Ebola viral load correlate with mortality and survival time in 288 Ebola virus disease patients. Int J Infect Dis. 2016;42:34–9. doi:10.1016/j.ijid.2015.10.021.
35. Matson MJ, Ricotta EE, Rollin PE, Bausch DG, Sanchez A, Crary SM, et al. Evaluation of viral load in patients with Ebola virus disease in Liberia: a retrospective observational study. Lancet Microbe. 2022 Jul;3(7):e533–e542.
36. Smither SJ, Lear-Rooney C, Biggins JE, Pettitt J, Lever MS, Olinger GG. Comparison of the plaque assay and 50% tissue culture infectious dose assay as methods for measuring filovirus infectivity. J Virol Methods. 2013 Nov;193(2):565–71. doi:10.1016/j.jviromet.2013.05.015
37. World Organisation for Animal Health (WOAH). World Animal Health Information System (WAHIS) [Internet]. 2024 [cited 2025 May 21]. Available from: https://wahis.woah.org/
38. U.S. Geological Survey (USGS). Wildlife Health Information Sharing Partnership – event reporting system (WHISPers) [Internet]. 2024 [cited 2025 May 21]. Available from: https://whispers.usgs.gov/
39. Wille M, Geoghegan JL, Holmes EC. How accurately can we assess zoonotic risk? PLoS Biol. 2021;19(4):e3001135. doi:10.1371/journal.pbio.3001135.
40. Gibb R, Albery GF, et al. Mammal virus diversity estimates are unstable due to accelerating discovery effort.
41. Mollentze N, Streicker DG. Viral zoonotic risk is homogenous among taxonomic orders of mammalian and avian reservoir hosts. Proc Natl Acad Sci U S A. 2020;117(17):9423–30. doi:10.1073/pnas.1919176117.
42. Weber N, Moya R, et al. Robust evidence for bats as reservoir hosts is lacking in most African virus studies. Biol Lett. 2023;19(4):20230358. doi:10.1098/rsbl.2023.0358.
43. Caron A, et al. Ebola virus maintenance: if not (Only) bats, what else? Viruses. 2018;10(10):pii: E549. doi:10.3390/v10100549.

44. Judson SD, Fischer R, Judson A, Munster VJ. Ecological contexts of index cases and spillover events of different ebolaviruses. PLoS Pathog. 2016;12(8):e1005780. doi:10.1371/journal.ppat.1005780.
45. Leroy EM, Kumulungui B, Pourrut X, Rouquet P, Hassanin A, Yaba P, et al. Fruit bats as reservoirs of Ebola virus. *Nature*. 2005 Dec 1;438(7068):575–6. doi:10.1038/438575a.
46. Gibb R, Albery GF, Mollentze N, Eskew EA, Schneider M, Ryan SJ, et al. Data proliferation, reconciliation, and synthesis in viral ecology. ***BioScience***. 2021 Nov;71(11):1148–56. doi:10.1093/biosci/biab080.
47. Monadjem A, Montauban C, Webala PW, Laverty TM, Bakwo-Fils EM, Torrent L, et al. African bat database: curated data of occurrences, distributions and conservation metrics for sub-Saharan bats. Sci Data. 2024 Dec 2;11(1):1309. doi:10.1038/s41597-024-04170-7.
48. Bennett RS, Logue J, Liu DX, Reeder RJ, Janosko KB, Perry DL, et al. Kikwit Ebola Virus Disease Progression in the Rhesus Monkey Animal Model. Viruses. 2020 Jul 14;12(7):753. doi:10.3390/v12070753.
49. Riesle-Sbarbaro SA, Wibbelt G, Kurth A. Selective replication and vertical transmission of Ebola virus in experimentally infected Angolan free-tailed bats. Nat Commun. 2024;15:925. doi:10.1038/s41467-024-45231-0.
50. Schuh AJ, Amman BR, Sealy TK, Kainulainen MH, Chakrabarti AK, Guerrero LW, et al. Antibody-mediated virus neutralization is not a universal mechanism of Marburg, Ebola, or Sosuga virus clearance in Egyptian rousette bats. J Infect Dis. 2018 Jun 1;219(11):1716–21. doi:10.1093/infdis/jiy733.
51. Miranda MEG. Seroepidemiological study of filovirus related to Ebola in the Philippines. Lancet. 1991;337:425. doi:10.1016/0140-6736(91)91199-5.
52. Leroy EM, Rouquet P, Formenty P, et al. Multiple Ebola virus transmission events and rapid decline of central African wildlife. Science. 2004;303(5656):387–90. doi:10.1126/science.1092528.
53. Leroy EM, Rouquet P, Formenty P, et al. A serological survey of Ebola virus infection in central African nonhuman primates. J Infect Dis. 2004;190(11):1895–9. doi:10.1086/425421.

54. Wittmann TJ, et al. Isolates of Zaire ebolavirus from wild apes reveal genetic lineage and recombinants. Proc Natl Acad Sci U S A. 2007;104(43):17123–7. doi:10.1073/pnas.0704076104.

55. Barrette RW, Metwally SA. Discovery of swine as a host for the reston ebolavirus. Science. 2009;325(5937):204–5. doi:10.1126/science.1172705.

56. Taniguchi S, Watanabe S, Masangkay JS, Omatsu T, Ikegami T, Alviola P, Ueda N, Iha K, Fujii H, Ishii Y, Mizutani T, Fukushi S, Saijo M, Kurane I, Kyuwa S, Akashi H, Yoshikawa Y, Morikawa S. Reston ebolavirus antibodies in bats, the Philippines. J Infect Dis. 1991;163(3):524–7. doi:10.1086/514314.

57. Schwantes CJ, Sánchez CA, Stevens T, Zimmerman R, Albery G, Becker DJ, et al. A minimum data standard for wildlife disease research and surveillance. Sci Data. 2025 Jun 21;12(1):1054. doi: 10.1038/s41597-025-05332-x.

Supporting information Captions

S1 Appendix - Summary of the metadata in the scientific papers referenced for Orthoebolavirus entries in the VIRION database

Figure 1

Click here to access/download;Figure;Figure 1 final.tiff

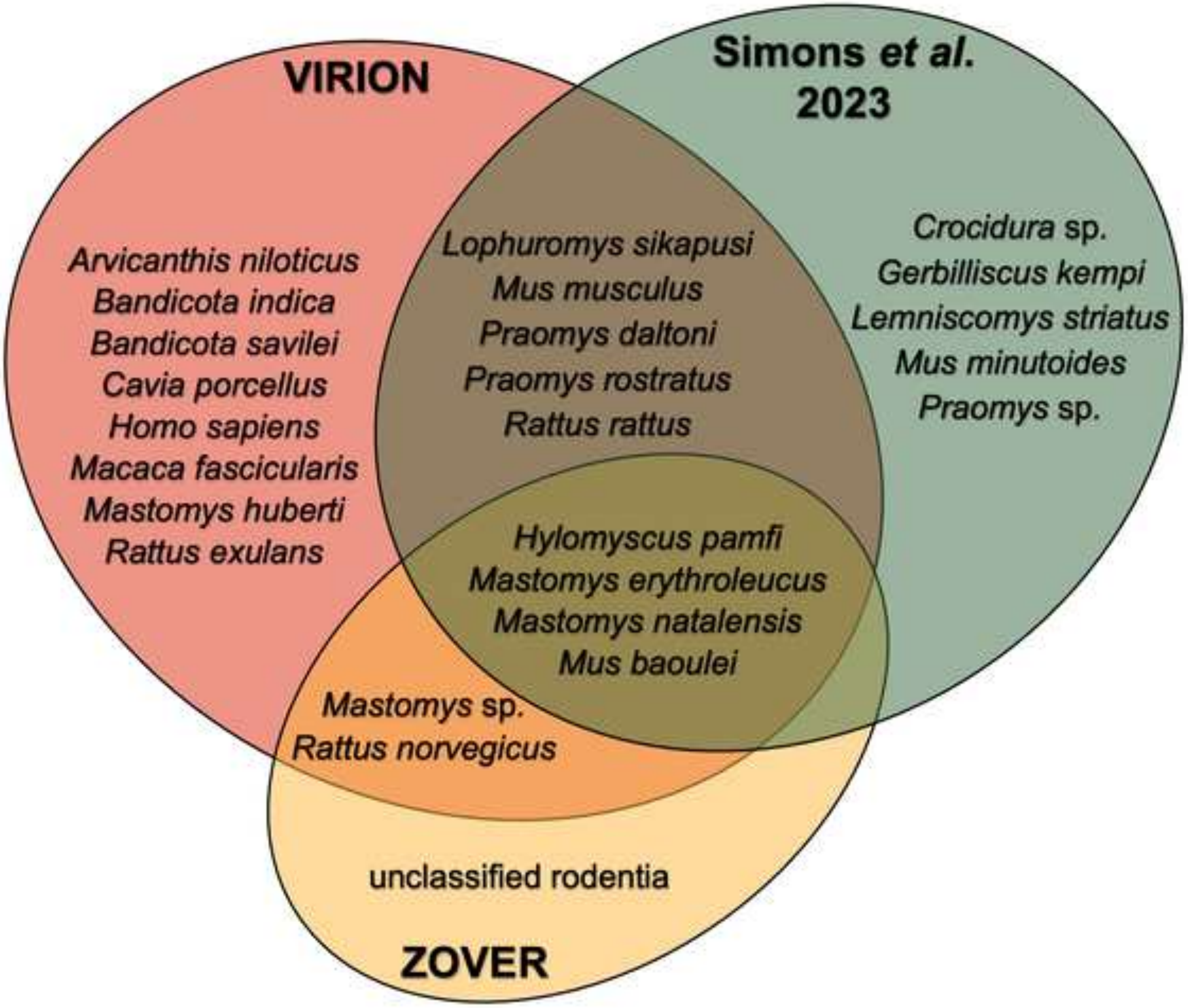

Figure 2

Click here to access/download;Figure;Figure 2 final.tiff

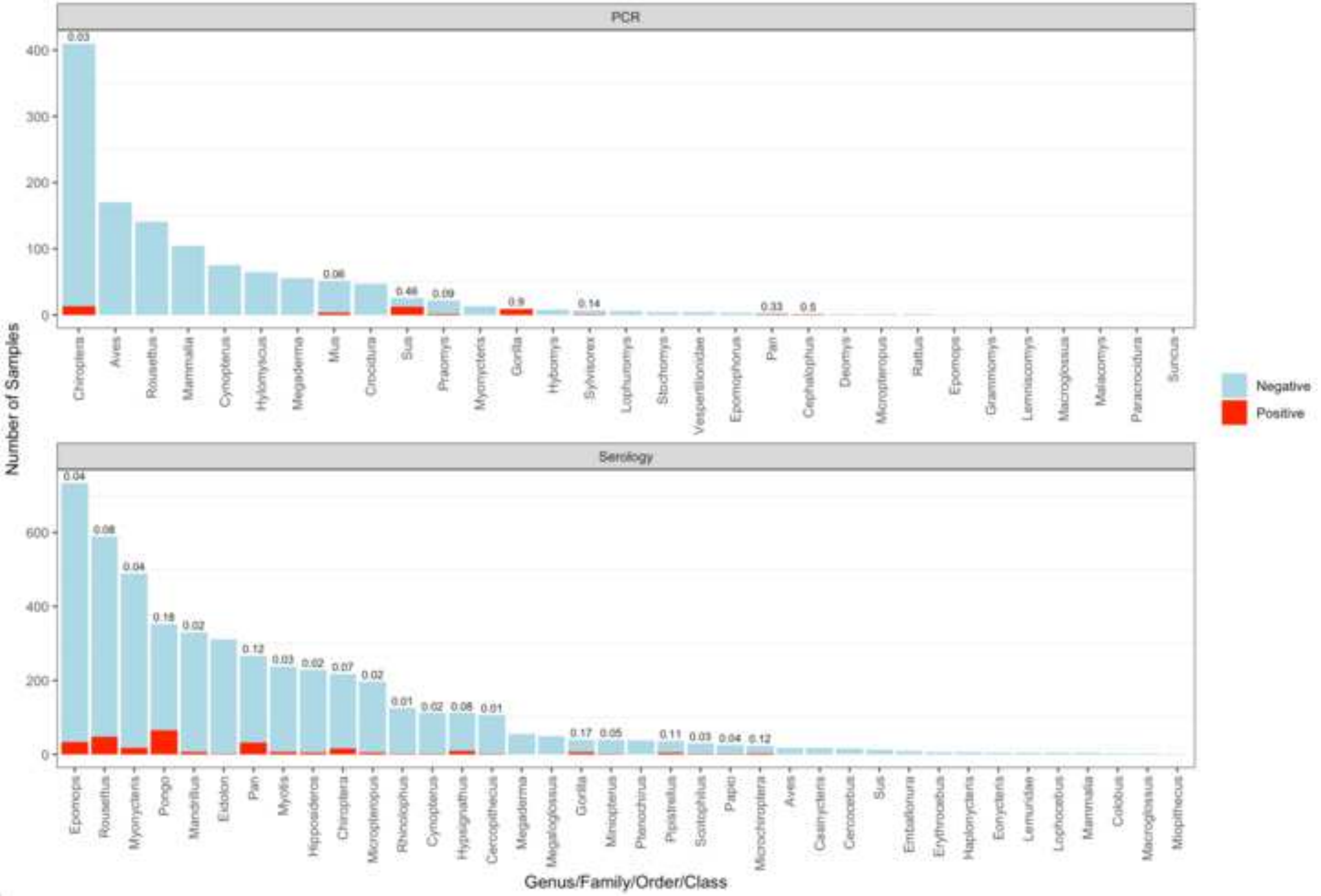

Figure 3

Click here to access/download;Figure;Figure 3 final.tiff

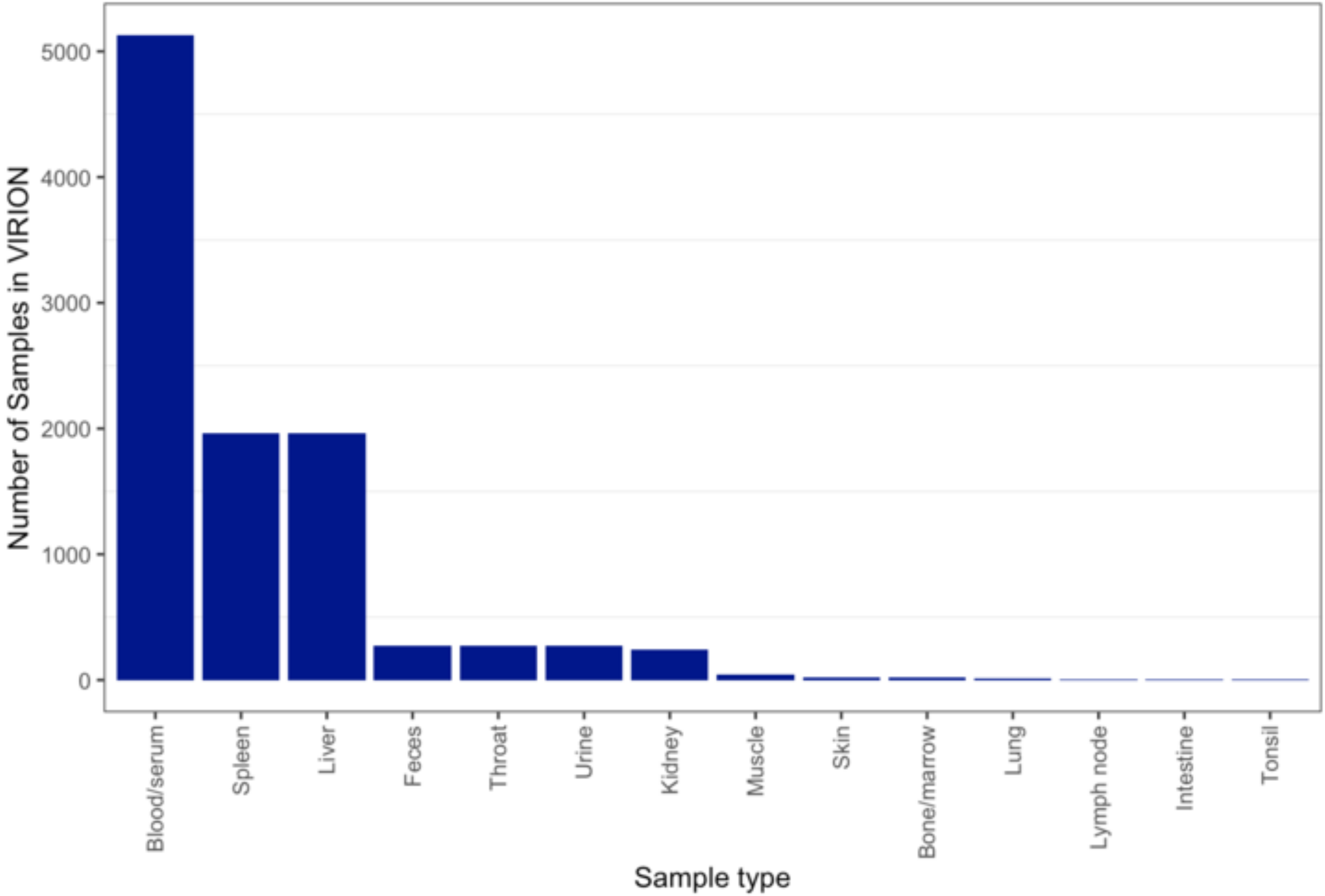

S1 Appendix

Click here to access/download

Supporting Information

Supporting Information_Imogen Lindsley.xlsx

<u>Rebuttal letter</u>

Article: " Host-virus association databases as tools for understanding viral spillover at varying scales"

We would like to begin by thanking the reviewers for their time spent on evaluating this manuscript. We explain how we have addressed the point raised below. The statement of the reviewer is presented in bold font.

1) **This appears to be a resubmission of the manuscript PNTD-D-25-01041. Please include a rebuttal letter with a response to the decision and reviewers' comments (if applicable), detailing any revisions made to the original submission. Please upload this as a 'Response to Reviewers' file type.**

We have resubmitted this manuscript (PNTD-D-25-01065) as a research article as when we submitted it as a review (under PNTD-D-25-01041) we received the following feedback:

Thank you for submitting your manuscript to PLOS Neglected Tropical Diseases. As with all papers, your manuscript was reviewed by members of the editorial board. Based on our assessment, we ask you to resubmit your article in the proper category, as an original article and not a review, since it includes a practical "experiment".

Therefore, we are now submitting the manuscript as a research article under the reference number: PNTD-D-25-01065. We have resubmitted it is a research article as it includes a practical 'experiment'.
